# Relationships between Membrane Binding, Affinity and Cell Internalization Efficacy of a Cell-Penetrating Peptide: Penetratin as a Case Study

**DOI:** 10.1371/journal.pone.0024096

**Published:** 2011-09-06

**Authors:** Isabel D. Alves, Cherine Bechara, Astrid Walrant, Yefim Zaltsman, Chen-Yu Jiao, Sandrine Sagan

**Affiliations:** 1 UPMC Univ Paris 06, UMR 7203, LBM, Paris, France; 2 CNRS, UMR 7203, LBM, Paris, France; 3 ENS, UMR 7203, LBM, Paris, France; Consejo Superior de Investigaciones Cientificas, Spain

## Abstract

**Background:**

Penetratin is a positively charged cell-penetrating peptide (CPP) that has the ability to bind negatively charged membrane components, such as glycosaminoglycans and anionic lipids. Whether this primary interaction of penetratin with these cell surface components implies that the peptide will be further internalized is not clear.

**Methodology:**

Using mass spectrometry, the amount of internalized and membrane bound penetratin remaining after washings, were quantified in three different cell lines: wild type (WT), glycosaminoglycans- (GAG^neg^) and sialic acid-deficient (SA^neg^) cells. Additionally, the affinity and kinetics of the interaction of penetratin to membrane models composed of pure lipids and membrane fragments from the referred cell lines was investigated, as well as the thermodynamics of such interactions using plasmon resonance and calorimetry.

**Principal Findings:**

Penetratin internalized with the same efficacy in the three cell lines at 1 µM, but was better internalized at 10 µM in SA^neg^>WT>GAG^neg^. The heat released by the interaction of penetratin with these cells followed the ranking order of internalization efficiency. Penetratin had an affinity of 10 nM for WT cells and µM for SA^neg^ and GAG^neg^ cells and model membrane of phospholipids. The remaining membrane-bound penetratin after cells washings was similar in WT and GAG^neg^ cells, which suggested that these binding sites relied on membrane phospholipids. The interaction of penetratin with carbohydrates was more superficial and reversible while it was stronger with phospholipids, likely because the peptide can intercalate between the fatty acid chains.

**Conclusion/Significance:**

These results show that accumulation and high-affinity binding of penetratin at the cell-surface do not reflect the internalization efficacy of the peptide. Altogether, these data further support translocation (membrane phospholipids interaction) as being the internalization pathway used by penetratin at low micromolecular concentration, while endocytosis is activated at higher concentration and requires accumulation of the peptide on GAG and GAG clustering.

## Introduction

Penetratin (H-RQIKIWFQNRRMKWKK-NH_2_) is a peptide derived from the third helix of the homeodomain of Antennapedia [1]–[5], whose internalization mechanism has been widely studied [6]–[12]. This peptide internalizes in all types of cells, however with varied efficiency. It has been reported that penetratin enters cells either by endocytosis [7], [13]–[15], or endocytosis and direct membrane translocation [16], [17]. Penetratin binds heparan sulfates [18]–[21] or chondroitin sulfates [21], [22] with high-affinity, which has led to support endocytosis as a unique internalization pathway for penetratin. However, using plasma membrane vesicles, penetration in the absence of endocytic processes was also recently reported for fluorescently labeled nona-arginine, Tat peptide, Penetratin, MAP, Transportan and TP10 [23].

With the use of model systems, the binding of penetratin to heparan sulfate proteoglycans (HSPGs) has been investigated using isothermal titration calorimetry [18]–[19] ESR spectroscopy [21] or fluorescence spectroscopy [20]. All these studies report that penetratin binds tightly to HSPGs such as heparan sulfates (HS), heparin and chondroitin sulfate (CS) with dissociation constants in the low micromolar range. Enhanced binding affinity has been observed in the presence of anionic lipids, both for penetratin and other CPPs, using a variety of different approaches [24]–[30]. Although a relation between the amount of CPP bound to the cell surface and its degree of internalization may seem intuitively obvious, this is currently not clear. Indeed while Drin et al. [31] have proposed that this may be the case and a very recent study shows a direct correlation between the binding of penetratin to and its uptake by plasma membrane vesicles (PMVs) [32], studies from our laboratory point to a lack of connection between cell surface high-affinity binding and uptake efficiency in the case of penetratin and other CPPs [33], [16]. Indeed, we had previously demonstrated that the internalization pathways of the most studied cell-penetrating peptides (Tat, R9, penetratin) were different according to the cell membrane composition [16]. These results implied that these peptides could recognize with different affinity numerous membrane components.

We have now further examined the internalization efficacy of penetratin as well as its macroscopic binding affinity to model and cell membranes of different lipid (zwitterionic, anionic and lipids with tendency to form negative membrane curvature) and carbohydrate composition (wild-type, GAG-deficient or sialic acid-deficient cells). Such studies were performed using a combination of both cellular and biophysical methods to allow a broader characterization.

## Results

### Quantity of internalized penetratin

**Figure 1:**
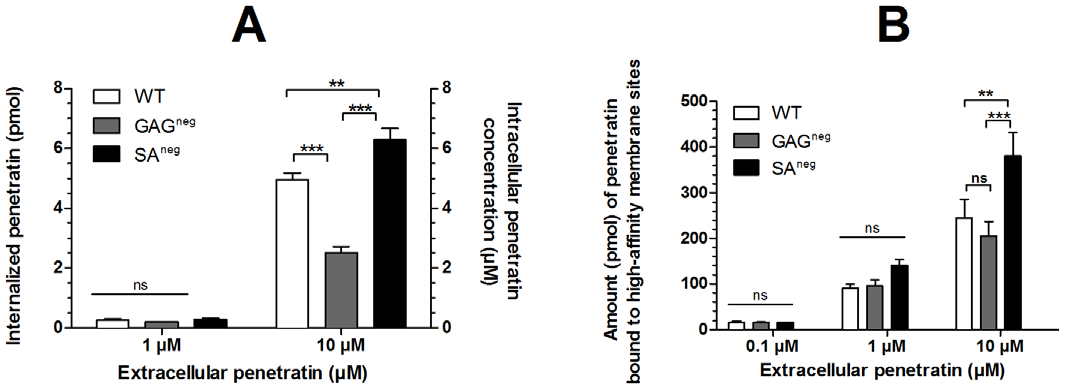
Amount (pmoles) of A) internalized and B) high-affinity membrane bound penetratin in WT, GAG^neg^ and SA^neg^ cells. Corresponding intracellular penetratin concentrations (µM) are also indicated (A) taken the volume of one cell as being 1 pL.

As already reported, the quantity or concentration of internalized and membrane-bound penetratin could be differentiated and measured by MALDI mass spectrometry [16], [33], [34]. This is one of the few methods that allows such distinction and uses a peptide possessing a biotin and glycine tag proven to be neutral in the process of membrane interaction which is often not the case when fluorescent tags are used [35], [36]. Using the reported protocol, the amount of internalized penetratin was quantified in one million cells that were incubated with 1 or 10 µM penetratin for one hour. After washing steps, the extracellular membrane-bound peptide was degraded by trypsin digestion [16], [33], [34] so that only the internalized peptide was quantified. Incubated at 10 µM with cells, penetratin internalized significantly more in WT and SA^neg^ than in GAG^neg^ cells, respectively (Figure 1A). However, with 1 µM penetratin, no significant difference was measured in the internalized peptide quantity, that is about 0.25 pmole (0.25 µM, provided a cell volume of 1 pL) in the three cell lines. The extracellular concentration could not be lowered further because of the lack of sensitivity of the MALDI-MS method for accurate measurements of sub-picomole quantities of penetratin.

### Cell surface binding of penetratin

#### Quantification of penetratin bound to high-affinity membrane-binding sites

After one-hour incubation with penetratin, the high-affinity membrane-associated peptide could be accurately measured by omitting the trypsin treatment of cells, as already demonstrated with permeant [16], [33] and weakly permeant [34] peptides, using the reported mass spectrometry protocol. Accordingly, the amount of penetratin bound to the cell surface, and thus having resisted the washing steps, was determined for the three cell lines incubated with different concentration of penetratin (Figure 1B). At 10 µM penetratin, a significant difference was found between the membrane quantity averages of WT and SA^neg^ (**P<0.01) and of GAG^neg^ and SA^neg^ cells (***P<0.001), but not between WT and GAG^neg^ cells. However at 1 and 0.1 µM extracellular concentration, the same amount of peptide-bound to high-affinity binding sites was found in the three cell lines. Thus, it appears that the membrane of WT and GAG^neg^ cells retained penetratin in a similar manner, while SA^neg^ cell membranes displayed a higher membrane binding capacity or greater number of binding sites at high peptide concentration.

To obtain information about the affinity (Kd), kinetics and thermodynamics of penetratin interaction with the cell surface, two biophysical approaches were used: a novel plasmon resonance method named plasmon waveguide resonance (PWR) and isothermal titration calorimetry (ITC). To be able to correlate membrane binding with membrane accumulation and internalization in cells and get insight into the role of membrane carbohydrates on peptide-membrane recognition, WT, GAG^neg^ and SA^neg^ CHO cells or cell membrane fragments were used in ITC and PWR, respectively, an approach that was quite innovative for such applications and that required a certain protocol optimization.

#### Affinity

**Figure 2:**
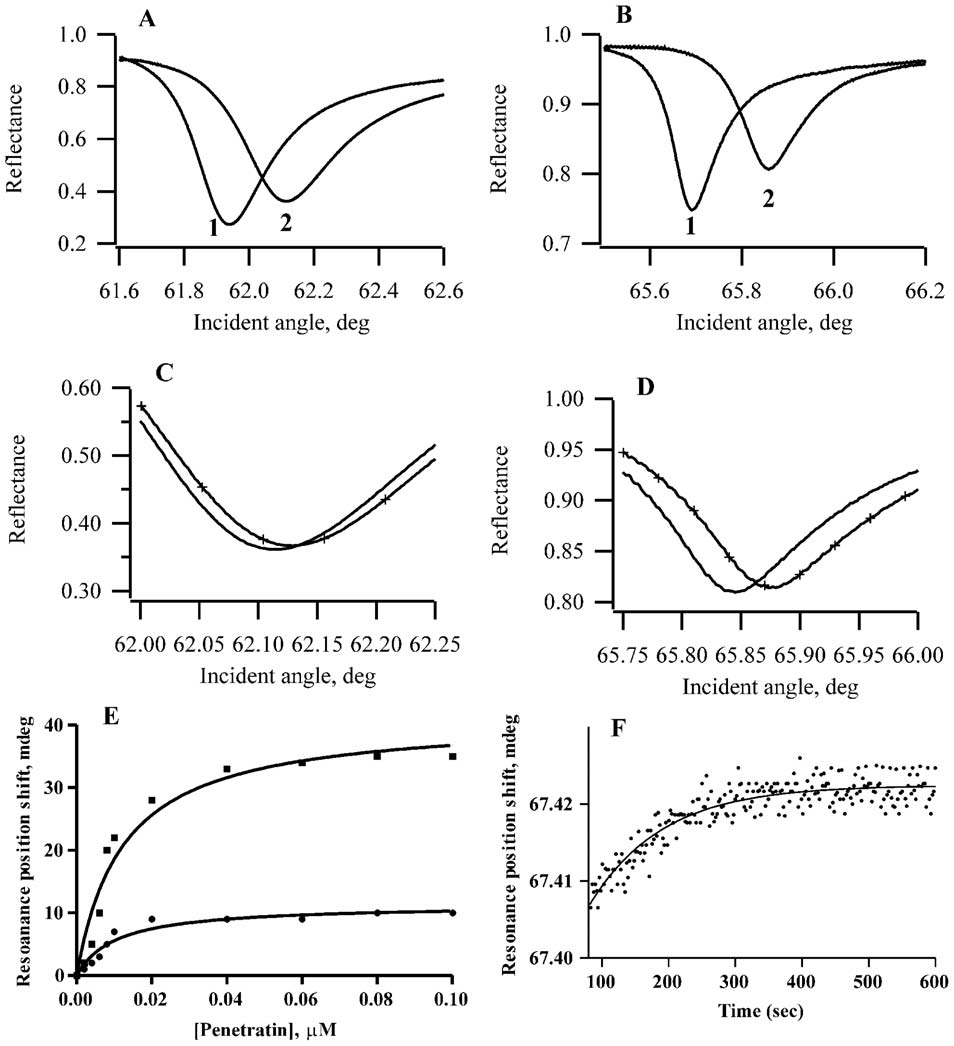
Formation of a lipid bilayer containing WT membrane fragments and penetratin interaction with this membrane monitored by PWR. Panels A) and B) correspond to the PWR spectra obtained for the buffer (1) and the lipid bilayer (2) and Panels C) and D) for spectra obtained after addition of about 0.1 µM of penetratin (+) to the bilayer (solid line) obtained for p- and s-polarized light, respectively. The resonance position shifts obtained for p- (•) and s- (▪) polarizations for the incremental addition of penetratin are represented in Panel E, together with the hyperbolic binding (affinity constants are provided in table 2). Panel F corresponds to the kinetic measurements obtained for the p-pol light upon addition of penetratin (0.05 µM) to the lipid bilayer (rate constants are presented in Table 3).

Homogenized membranes were obtained by osmotic and mechanical disruption of cells [37]. A method to deposit cell membrane fragments on the PWR cell sample was developed as follows. The deposition of a membrane composed of cell membrane fragments into the PWR resonator led to spectral changes, namely large positive spectral shifts for both *p*- and *s*-polarizations relative to the buffer spectrum (Figure 2, panels A and B) indicating the immobilization of a significant amount of mass on the sensor surface. It should be noted that the shifts obtained for *p*-pol were considerably larger than the ones obtained for *s*-pol (s/p ratio about 0.7). This indicated that bilayer deposition was accompanied by a strong anisotropic change with a larger mass change occurring perpendicular than parallel to the cell surface. Such anisotropic spectral changes correlated well with published results for deposition of planar bilayers composed of model lipid systems, indicating that lipids were not randomly distributed on the sensor surface but were at least partially oriented with their long axis perpendicular to the sensor surface.

To confirm that planar lipid bilayers prepared by the method above described had incorporated cell membrane fragments, the response of planar lipid bilayers composed of cell membrane fragments containing the NK-1 receptor to substance P, a classic agonist for the NK-1 receptor, was monitored. The interaction of substance P with the membrane led to positive spectral shifts for both *p*- and *s*-polarizations. Since no effect was induced by the interaction of substance P with lipid bilayers that lacked NK-1 receptor; such spectral changes were related to NK-1 receptor conformational changes. Moreover, a binding affinity (determined by monitoring the spectral changes for incremental ligand addition and plotting the binding isotherm) of about 1 nM was obtained, which was in good agreement with that determined using reconstituted NK-1 receptor into planar lipid model bilayers by PWR [38]. The results were reproducible on three sets of independent experiments. This indicated that the prepared lipid bilayers have incorporated in the correct leaflet orientation, at least some of the cell membrane fragments in the correct leaflet orientation. It was not possible to exclude that part of the incorporated membrane fragments was exposing the inner cell membrane leaflet, or was contaminated with organelles membrane (Golgi, ER).

The addition of penetratin to the PWR cell sample containing cell membrane fragments led to spectral shifts in both *p*- and *s*-polarization indicating that a change in mass and organization in the lipid bilayer occurs upon penetratin contact (Figure 2). Regarding the direction of the PWR spectral shifts, two types of behavior were observed depending on the membrane composition: 1) in the case of GAG^neg^ cells, penetratin led to a two phase binding process with negative shifts followed by positive shifts (for both polarizations) which equilibrated after certain concentrations; 2) in the case of WT and SA^neg^ cells, penetratin led to a single binding process with positive shifts for both polarizations (see panels C and D in Figure 2 for penetratin interaction with WT cell membranes and Table 1 for the direction and magnitude of the spectral shifts). This indicated that different penetratin-induced lipid reorganization was taking place in the two different scenarios, a point that is further developed below. Penetratin affinity to lipid bilayers and membrane fragments could be calculated using PWR by measuring the resonance minimum position shifts upon incremental addition of peptide to the cell sample [39]. Applying such latter method, the presence of carbohydrates in the cell membrane surface greatly influenced the peptide affinity: a Kd of 0.01 µM was observed for the interaction of penetratin with WT membrane fragments while a lower affinity existed for cell membrane fragments lacking SA or GAG, which was 0.3 and 2 µM, respectively (Table 2). The possibility of different leaflet orientation of membrane fragments cannot be ruled out. As the inner membrane leaflet was likely similar for the three cell types, differences that were observed in terms of binding affinity could be attributed to the differences in the composition of the outer membrane leaflet, that relied principally on the presence and distribution of carbohydrates at the cell-surface. Indeed, it has been previously shown that the addition of glycosaminoglycans to the core proteins was not a prerequisite for cell surface expression of these proteoglycans [40]–[42]. Thus, it was assumed that the major molecular modification of the membrane that penetratin encountered, was the difference in carbohydrate composition between the three cell lines.

**Table 1:** Direction, magnitude and graphical analysis of the spectral changes observed by PWR upon penetratin interaction with the membrane composed of cell membrane fragments.

Lipid System	WT		GAG^neg^	SA^neg^
Number of binding events	1		2	1
Spectral Shifts (mdeg)				
*p* polarization	+10	−5	+15	+8
*s* polarization	+35	−5	+18	+28
Mass/structural changes (%)	60/40	100/0	83/17	63/37

**Table 2:** Affinity between penetratin and lipid bilayers composed of cell (WT, GAG^neg^, SA^neg^) membrane fragments; data obtained with the PWR technique.

Lipid System		Kd (µM)
Cell Membrane Fragments	WT	0.01±0.003
	GAG^neg^	2.0±0.6
	SA^neg^	0.3±0.04

Note: the experiments were repeated 3 times.

#### Penetratin-induced lipid reorganization

Aside from the affinity constants between the peptide and the membrane, PWR also provided information on peptide and lipid reorganization following their interaction (Table 1). Two data analysis approaches gave different information: the optical parameters obtained from fitting procedures (refractive indices and extinction coefficients for both *p*- and *s*-polarizations (*n_p_* and *n_s_*; *k_p_* and *k_s_*) and membrane thickness *t*) provided information about mass density in the immobilized film, in addition to molecular anisotropy reflecting the orientation relative to the membrane plane and hence information about conformation [43]–[48]; a graphical analysis characterized the mass and the structural changes that accompanied the interaction of the peptide with the lipid bilayer [46]. Herein, we have chosen to perform a graphical analysis, in which plotting the data points on a *(s*, *p*) coordinate system containing both mass and structural axis placed according to the sensitivity factor of the PWR sensor (see Materials and Methods for details), allowed the determination of the mass and structural anisotropy contributions to the process (Table 1). For the membrane interaction where two binding processes were observed, as in the case of GAG^neg^, the first binding, named high affinity binding process because it occurred at lower penetratin concentrations, was associated with negative spectral shifts for both *p*- and *s*-polarizations, which were totally attributed to mass changes. As for the second event, this low affinity binding process was associated with positive spectral shifts for both polarizations with 83% of the spectral changes due to mass changes (mass increase in this case because the spectral shifts were positive) and 17% resulting from structural changes.

Such biphasic behavior was not observed for the interaction of penetratin with membranes composed of WT and SA^neg^ membrane fragments, where only positive shifts were observed.

#### Kinetics

The kinetics of penetratin interaction with the membrane was determined by following the shift in the resonance minimum position as a function of time for a peptide concentration slightly above the Kd value. The interaction of penetratin with cell fragment membranes led to a small decrease of the resonance minimum position, followed by a plateau where the signal was only slightly fluctuating. The plateau was followed by an increase in the resonance angle position, which then stabilized (Figure 2, panels E and F). This first plateau was more or less comparable between the different membranes and was of about 100 seconds. Rate constants were determined for the second part of the signal (the resonance angle increase) and varied upon membrane composition (Figure 2 and Table 3). As one can see in Table 3, the rate constants observed for the different membranes varied slightly, with the following order starting with the faster: SA^neg^>GAG^neg^>WT CHO cells.

**Table 3:** Kinetic analysis of the interaction of penetratin with lipid membranes; data obtained by PWR spectroscopy.

Lipid	EggPC	EggPG	EggPC/DOPE	Egg PC/POPG	WT	GAG^neg^	SA^neg^
10^3^ k (s^−1^)	30±2	90±4	50±3	80±3	9±0.6	10±0.9	20±1

Note: experiment was repeated 3 times.

#### Thermodynamics

**Figure 3:**
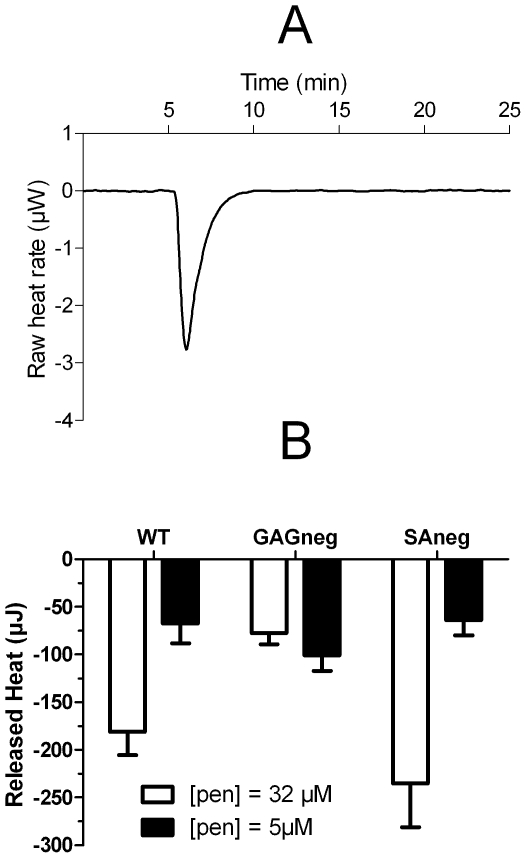
(A) Isothermal calorimetric titration of 50 nmol penetratin to a suspension of 1.5 million CHO cells at 37°C. (B) Released heat measured after the addition of 50 nmol (white, final concentration 32 µM) and 7.75 nmol (black, final concentration 5 µM) penetratin to WT, GAG^neg^ and SA^neg^ cells.

Isothermal titration calorimetry (ITC) was extensively used to study the binding of membrane active peptides to model lipid systems such as lipid large unilamellar vesicles [18], [19], [27]. In contrast, calorimetry has been much less exploited to study the interaction of molecules with living cells, mainly because cells become anoxic due to the lack of oxygen within the time course of the experiment [47]. However, one study has reported the use of ITC to characterize the thermodynamics of a peptide binding to whole cells [48]. Howe and collaborators [48] measured the heat produced by the antimicrobial peptide polymixin B binding to different bacterial strains, and showed it could be related to the bacteria's sensitivity against the drug. In the present study, we compared the heat produced by the binding of 50 nmol or 7.75 nmol penetratin (corresponding to extracellular concentrations of 32 µM and 5 µM respectively) WT, GAG^neg^ and SA^neg^ cells, within a short time-course assay. In terms of peptide/lipid ratio, the use of 5 and 32 µM extracellular concentrations with the cells in suspension could be assimilated to 1 and 10 µM concentrations applied to adherent cells, respectively [17], [49]. It should be noted that due to the limitations in the method regarding sensibility (heat release), one could not use peptide concentrations in the order of the magnitude of the Kd value for the WT (0.01 µM) and SA^neg^ (0.3 µM) cells. The addition of penetratin to the cells always led to an exothermic reaction for the two studied concentrations (Figure 3A). The heat releases were different according to the cell lines and extracellular concentrations. The addition of 50 nmol penetratin to cells resulted in a heat release of −240±50 µJ for SA^neg^ cells, −180±30 µJ for WT cells and only −80±10 µJ for GAG^neg^ cells (Figure 3B). On the other hand, the addition of 7.75 nmol penetratin led to smaller heat releases, with less differences between the three cell lines: −70±20 µJ for WT cells, −60±20 µJ for SA^neg^ cells and −100±20 µJ for GAG^neg^ cells (Figure 3B).

### Model membrane binding of penetratin: affinity, lipid reorganization and kinetics

#### Affinity

To get some insight into the role of the lipid component in the cell surface regarding penetratin interaction, lipid model systems composed of varied lipid composition were used. Both zwitterionic (egg PC), anionic (egg PG -phosphatidylglycerol-and egg PC/DOPG -phosphatidylglycerol/dioleoylphosphatidylglycerol-) and lipids with propensity to form negative membrane curvature (egg PC/DOPE -dioleoylphosphatidylethanolamine-) were chosen in order to understand the role of electrostatic interactions and membrane curvature in the interaction of penetratin with the lipid bilayer. For all the lipid systems, penetratin interaction led to spectral shifts in both the *p*- and *s*-polarisation. As in the case of cell membrane fragments studies, presented above, two distinct responses were observed depending on the membrane composition: 1) in the case of egg PC and egg PC/DOPE, a two phase binding process with negative shifts followed by positive shifts (for both polarizations) which equilibrated after certain concentrations were observed; 2) in the case of egg PG, egg PC/POPG (1-palmitoyl-2-oleoyl-sn-glycero-3-phospho-(1′-rac-glycerol)), penetratin led to a single binding process with positive shifts for both polarizations (see Table 1 for the direction and magnitude of the spectral shifts). Only positive shifts were observed in the case of WT and SA^neg^ cell membrane fragments. Interestingly, all these membranes share the property of being rich in negatively charged molecules (either anionic lipids or carbohydrates). The affinity of penetratin to the membrane was highly dependent on lipid composition, while with egg PC a 28 µM affinity was observed, a low to sub-micromolar affinity existed in the presence of phosphatidylglycerol or phosphatidylethanolamine (Table 4). The electrostatic interaction played an important role in penetratin interaction with the membrane, but was not the only relevant factor, the shape of the lipid headgroup and/or the lipid curvature seemed to also play a key role in (this will be further discussed below).

**Table 4:** Affinity between penetratin and lipid bilayers of synthetic lipids; data obtained with the PWR spectroscopy technique.

Lipid bilayer	Kd (µM)
Egg PC	28±2
Egg PG	0.3±0.02
Egg PC/DOPE	0.5±0.01
Egg PC/DOPG	1.4±0.03

Note: the experiments were repeated 3 times.

#### Lipid reorganization

For the membrane interaction where two binding processes were observed, as in the case of egg PC, egg PC/DOPE, the first binding was associated with negative spectral shifts for both *p*- and *s*-polarizations, which corresponded mainly to mass changes (90–100%). As for the second event, the low affinity binding process was associated with positive spectral shifts for both polarizations with 80–90% of the spectral changes due to mass changes (mass increase in this case because the spectral shifts are positive) and 10–20% resulting from structural changes. In the case where only one binding event was observed (egg PG and egg PC/DOPG) mass changes again dominated the spectral changes (85 to 91%) (Table 5).

**Table 5:** Direction, magnitude and graphical analysis of the spectral changes observed by PWR upon penetratin interaction with the synthetic lipid model membrane.

Lipid System	Egg PC		Egg PG	Egg PC/DOPE(1/1)		Egg PC/POPG(3/1)
Number of binding events	2		1	2		1
Spectral Shifts (mdeg)						
*p* polarization	−15	+22	+30	−8	+13	+25
*s* polarization	−14	+28	+33	−9	+9	+28
Mass/structural changes (%)	90/10	80/20	85/15	100/0	90/10	91/9

#### Kinetics

Penetratin interaction and peptide-induced membrane reorganization occurred faster with synthetic membranes than with cell fragment membranes (Table 3). This might be related to the fact that there were more molecular rearrangements taking place in cell membranes due to the additional presence of carbohydrates and proteins when compared to synthetic membranes. There was a considerable faster response to penetratin for membranes containing anionic lipids.

In the process of penetratin binding to the membrane, two stages could be conceived: the first one corresponding to a simple contact with the membrane (either carbohydrates or lipid headgroups), and a second one where the peptide might have changed its localization and structure accompanied by lipid reorganization. Even though both steps should depend on the nature of the lipid, it was expected that the first step would be highly dependent on the presence of favorable peptide-membrane interactions such as electrostatic ones. This was most probably the reason why the kinetics was faster for egg PG (or egg PC/POPG 1/4) than for egg PC interaction. In the case of cell membrane fragments, the reduction in the rate constant may be correlated to a slow lipid and peptide reorganization after the first rapid binding step, overall giving a slower event. In the case of the bilayer containing PE, an increase in affinity was observed (when compared to a egg PC bilayer), and also an increase in the rate of membrane interaction. These results suggested that the presence of this phospholipid entailed a decrease in the dissociation rate of penetratin.

## Discussion

In this study we examined how lipid model and cell membranes of different composition influenced the affinity of penetratin, in relation with the internalization efficiency of the peptide.

At high micromolar concentration, penetratin internalized more in WT and SA^neg^ cells than in GAG^neg^ cells, while no significant difference in the internalized quantity was measured among cell lines for low µM of the peptide. These concentrations were chosen because we previously determined that they correspond to concentrations at which only translocation (membrane phospholipid dependent process) (1 µM) or translocation and endocytosis through GAG clustering (10 µM) occurred [16], [17]. The same significant differences in heat releases were observed among the different cell lines interacting with the high micromolecular peptide concentration, whereas these differences were dimmed for the low micromolar extracellular concentration. The heat produced by the binding of penetratin to the cell surface should be related to the number of peptides interacting with these sites or other phenomena induced by the binding of penetratin such as GAG clustering. The observed heat release was the sum of the heats of binding of penetratin to each site and subsequent phenomena; previous studies [18], [27], [50] indeed showed that the binding of cell penetrating peptides to phospholipid vesicles and heparan sulfate was an exothermic process. At low extracellular penetratin concentrations, little differences were observed between the three cell lines. However, at higher extracellular concentrations, the difference was clear. In wild type cells, all the potential binding sites (i.e. phospholipids and GAGs) were present on the cell surface. In GAG^neg^ cells, the observed heat release is twofold smaller than in the case of WT cells, showing that GAGs greatly contributed to the binding of penetratin to the cell surface. Also, in a previous study we suggested that at low concentrations, penetratin bound “passively” to GAGs, whereas higher peptides concentrations triggered GAG clustering [16] which could be the origin of the high heat release here. These results showed that at low micromolar concentration of penetratin, the three cells lines interacted with and internalized the peptide to the same extent. In contrast, differences in interaction and internalization were observed at high micromolar concentrations.

When examining the membrane affinity of penetratin to the three cell lines, it was obvious that the presence of carbohydrates in the cell membrane surface greatly influenced the peptide affinity. Greater intermolecular forces were indeed involved between penetratin and wild type membranes, than between the peptide and membranes lacking SA or GAG. Thus, from the ranking order of binding affinities, the internalization efficiency of penetratin should have been WT>SA^neg^>GAG^neg^. This was not the case since at a concentration above dissociation constants of penetratin for cell membranes (10 µM), the internalization efficiency of the peptide decreased from SA^neg^>WT>GAG^neg^. Therefore, these results showed that the affinity of the peptide for binding sites at the cell surface does not predict the efficacy of the peptide to be internalized. This conclusion could be applied to other cell-penetrating peptides [34], [50], [51], showing that it is not restricted to penetratin and should be a general rule.

The experimental data also underlined that penetratin had an affinity for cell membranes lacking GAG or SA similar to its affinity for pure phospholipid model membrane, in the micromolar range. There are several potential binding sites for penetratin at the cell surface, which include membrane lipids, glycosaminoglycans and sialic acids. In the case of SA, these negatively charged carbohydrates seemed to limit binding of penetratin to cells, since the absence of SA allowed the peptide to bind more membrane sites and to internalize more efficiently. As the membrane of the WT and GAG^neg^ cells, according to penetratin concentration, retained the peptide in similar quantities, it is likely that the binding sites that resisted washings mostly relied on peptide interactions with membrane phospholipids. This hypothesis would explain why the recent data reported by Amand and collaborators appeared to these authors contradictory to our previous results [32]. Using plasma membrane vesicles, the quantity of membrane-bound fluorescent penetratin was determined after centrifugation and found related to the quantity of internalized peptide in those vesicles [32]. In those experiments, there was no washing step. Therefore the membrane binding that was measured is not what was called here and in previous reported studies “high-affinity binding sites”, meaning binding sites that resisted washings [33], [16]. Interestingly, the data obtained with PMVs and fluorescence [32], were closely related to the ITC data obtained here, showing that the heat release followed up the internalization efficacy. In general, the notion of high affinity binding is implying a longer residence time for the ligand at its binding site than in the case for low affinity binding. In terms of binding capacity (dissociation constants), penetratin has more affinity for carbohydrates-containing membranes than for model membrane of pure phospholipids, although the presence of additional lipids in biological membranes might increase this affinity. Thus, it can be assumed that the association/interaction of penetratin with phospholipids is less reversible than with carbohydrates. Finally, it is interesting to notice that previous studies on vesicles have shown that the affinity of cell-penetrating peptides for phospholipid membranes was related to their capacity to translocate [52], [53], [36]. The situation was obviously more complicated with cells [54], [30], at the surface of which different sets of molecular partners are in competition to interact with cell-penetrating peptides. As shown herein, the global affinity of the peptides for the cell membrane does not reflect their internalization capacity.

In terms of the peptide-induced lipid reorganization, two types of behavior were observed depending on the membrane composition: 1) in the case of egg PC, egg PC/DOPE and GAG^neg^ cells, penetratin led to a two phase binding process with negative shifts followed by positive shifts (for both polarizations) which equilibrated after certain concentrations; 2) in the case of egg PG, egg PC/POPG, WT and SA^neg^ cells, penetratin led to a single binding process with positive shifts for both polarizations.

In case 1, the negative shifts were associated with a decrease in the mass density in the lipid bilayer, which could only be explained by an efflux of lipid out of the bilayer and into the plateau Gibbs border. This might be a consequence of the repulsion between the positive charges in penetratin and those in the phospholipid headgroups, as has been proposed earlier [25], [39]. The positive shifts observed at higher peptide concentration would result from a restructuring and reorganization of both peptide and lipid with higher packing responsible for the mass increase observed.

Such biphasic behavior was not observed for the interaction of penetratin with membranes composed of egg PG, egg PC/POPG and WT and SA^neg^ membrane fragments, where only positive shifts were observed (case 2). Interestingly, all these membranes shared the property of being rich in negatively charged molecules (either anionic lipids or carbohydrates). In this case, when comparing with zwitterionic lipid membranes, no repulsion could arose from the positive charges in the phospholipids and the peptide, but rather attraction forces between the positive charges in the peptide and the negative charges in the lipid came into play (discussed above).

If one compares mass and structural changes occurring in lipid model membranes *vs* cell membrane fragments, in the case of egg PG and egg PC/POPG mass changes dominated (85–91%), while in the case of WT and SA^neg^ cell fragments membranes they contributed to only about 60% of spectral changes. This indicates that, independently of the presence of negative charges (either from the lipid or the carbohydrates) penetratin induced different lipid reorganization in bilayers that possessed carbohydrates on their surface from those that did not. The predominance of structural changes in detriment of mass changes in the case of cell fragment membranes (as opposed to synthetic membranes), might be related to the fact that penetratin interaction occurred more superficially in membranes containing carbohydrates that did not penetrate between the fatty acid chains and thus did not have much lipid movement in and out of the plateau Gibbs border, which is mainly responsible for mass changes.

A comparison of PWR spectral shifts obtained with *p*- and *s*-pol provided insight into orientation of anisotropic molecules. Most circular dichroism studies have reported that penetratin adopts a α-helical structure in the presence of lipids (mainly anionic lipids), therefore an anisotropic structure. Herein, overall, the spectral shifts obtained with *s*-pol were larger than those obtained with *p*-pol; larger spectral changes parallel to the lipid membrane were expected if the peptide lied with its long axis parallel to the lipid membrane, as reported [55]–[57].

In conclusion, these data established PWR as a powerful technique to study the affinity of cell-penetrating peptides for biological membranes, and ITC as a relevant tool for screening cell-penetrating peptides interaction with cell membrane components that are potentially active in peptide internalization.

These data also demonstrated that the high binding affinity of penetratin to cell membrane carbohydrate components did not indicate whether those interacting partners were competent for internalization of the peptide. It was also strongly suggested that penetratin binding to phospholipids was less reversible than to carbohydrates, although the binding affinity of the peptide was greater in the latter case. Thus, at low micromolar concentrations of penetratin, carbohydrates can trap the peptide, leading to high local concentrations of the peptide (as already suggested for antimicrobial peptides [58]) that could interact with the phospholipid bilayer. Altogether, these data further supported translocation (membrane phospholipids interaction) as being the internalization pathway used by penetratin at low micromolecular concentration while endocytosis is activated at higher concentration and requires accumulation of the peptide on GAG and GAG clustering.

## Materials and Methods

One should note here that due to the inherent limitations associated with the sensitivity of each method used, the amounts of peptide used and/or P/L ratios used could not always be kept similar. This will be pointed out along the manuscript.

### General Methods

Egg PC was purchased from Avanti Polar Lipids (Alabaster, AL). Biot-(^1^H-Gly)_4_-RQIKIWFQNRRMKWKK-CONH_2_ and Biot-([2,2-^2^H]-Gly)_4_-RQIKIWFQNRRMKWKK-CONH_2_ were obtained from PolyPeptide Laboratories (Strasbourg, France). H-RQIKIWFQNRRMKWKK-CONH_2_ was synthesized using the Boc-solid phase strategy.

### Cell Culture

Wild type Chinese Hamster Ovary CHO-K1 cells (WT), xylosetransferase- or GAG-deficient CHO-pgsA745 [59] cells (GAG^neg^) and sialic acid deficient CHO-lec2 [60] cells (SA^neg^) were cultured in Dulbecco's modified Eagle's medium (DMEM) supplemented with 10% fetal calf serum (FCS), penicillin (100,000 IU/L), streptomycin (100,000 IU/L), and amphotericin B (1 mg/L) in a humidified atmosphere containing 5% CO_2_ at 37°C.

### Measure of cellular uptake and quantification of membrane-bound peptide

Cellular uptake was quantified using the method described by Burlina et al. [33]. In this protocol, the studied peptide bears a tag composed of four glycine residues together with a biotin moiety for purification purposes. After one-hour incubation with the peptide and washing, a protease is added (0.05% trypsin in Tris-HCl 100 mM pH 7.5) in order to detach the cells and to degrade the membrane-bound peptide. This avoids overestimating the quantity of internalized peptide due to the presence of peptides attached to the outer leaflet of the membrane. The cells are then lysed (0.3% Triton) and boiled, and the cell lysate is incubated with streptavidin-coated magnetic beads to extract the peptide from the lysate. For membrane-bound peptide quantification, washings but no protease digestion was done and the cells were directly lysed. The peptides are eluted from the streptavidin-coated magnetic beads with a α-cyano-4-hydroxycinnamic acid matrix and spotted on the MALDI plate. Mass spectrometry is not a quantitative method *per se*, therefore an internal standard is added to the lysis solution. The internal standard is a peptide that has the same sequence as the one being quantified except that it bears a tag composed of four ^2^H-containing instead of ^1^H-containing glycine residues. This allows the absolute quantification of internalized and membrane-bound peptide. The samples were analyzed by MALDI-TOF MS (positive ion reflector mode) on a Voyager DEPRO mass spectrometer (Applied Biosystems). The experiments were repeated at least three times independently.

### Isolation of cell membrane fragments

CHO cells of wild-type (WT), carbohydrate-modified (GAG^neg^ and SA^neg^) or expressing the NK-1 receptor [61] were grown to confluence in 225 cm^2^ tissue culture flasks. Confluent monolayers of cells were washed two times with 20 mL of 50 mM Tris-HCl buffer at pH 7.5 containing 150 mM NaCl and 2 mM EDTA and harvested using 10 mL of the same buffer. After centrifugation (1500 rpm for 15 min at 4°C), the pellet containing the cells was resuspended in 10 mM Tris-HCl buffer at pH 7.5 containing 1 mM EDTA and protease inhibitor mixture (Roche) and incubated for 1 h to induce cell rupture by osmotic shock. Disrupted cell membranes were then homogenized by 10 strokes with a tissue grinder. The homogenates were centrifuged at 1500 rpm for 10 min at 4°C to remove nuclei and cell debris and the supernatant collected. Supernatant was centrifuged at 20.000 rpm for 30 min and the membrane-enriched pellets were resuspended in 10 mM Tris-HCl pH 7.6, 0.1 M NaCl, 2 mM EDTA.

### Plasmon Waveguide Resonance (PWR) spectroscopy and formation of a planar lipid bilayer composed of cell membrane fragments

PWR spectra are produced by resonance excitation of conduction electron oscillations (plasmons) by light from a polarized CW laser (He-Ne; wavelength of 632.8 and 543.5 nm) incident on the back surface of a thin metal film (Ag) deposited on a glass prism and coated with a layer of SiO_2_ [43]–[45]. Experiments were performed on a beta PWR instrument from Proterion Corp. (Piscataway, NJ) that had a spectral resolution of 1 mdeg. The sample to be analyzed (a lipid bilayer membrane) was immobilized on the resonator surface and placed in contact with an aqueous medium, into which peptides can be introduced. Usually, the method used to prepare the planar lipid bilayer for PWR studies is the following: a small amount of lipid solution (of 8 mg/mL of lipid in butanol/squalene 0.93/0.07 v/v) is injected into the orifice in a teflon block separating the silica surface of the PWR resonator from the aqueous phase. Spontaneous bilayer formation is initiated when the sample compartment was filled with aqueous buffer solution (the method is based on the Montal-Mueller method to make black lipid membranes across a small hole in a teflon block and has been deeply described [39], [43]–[46]). Herein, the method was slightly changed to introduce cell membrane fragments as follows: after spreading the lipid solution (composed of egg PC) across the hole in the Teflon block, the sample compartment was filled with the membrane fragment solution obtained from cells (described in the previous paragraph). The molecules (such as lipids and peptides) deposited onto the surface plasmon resonator change the resonance characteristics of the plasmon formation and can thereby be detected and characterized. PWR spectra, corresponding to plots of reflected light intensity versus incident angle, can be excited with light whose electric vector is either parallel (*s*-polarization) or perpendicular (*p*-polarization) to the plane of the resonator surface. Spectral simulation [43]–[45] and/or graphical analysis [46] allow one to obtain information about changes in the mass density, structural asymmetry, and molecular orientation induced by bimolecular interactions occurring at the resonator surface. Here, the graphical analysis method was employed. Briefly this method consists in the deconvolution of the components of the PWR spectra that are due to changes in mass in the lipid film from those that are due to changes in structural anisotropy. Such distinction can be done based on the magnitude and direction of the PWR spectra shifts observed for the *p*- and *s*-polarized light. Thus, alterations in mass density (due to addition or subtraction of mass from the membrane) result in shifts in *p*- and *s*-polarization with the same magnitude and direction (isotropic changes), whereas structure alterations lead to anisotropic changes (shifts for *p*- and *s*-pol that are distinct in magnitude and direction). By plotting the spectral changes observed in a (*s*, *p*) coordinate system where mass (Δm) and anisotropy (Δstr) axes are represented based on the PWR sensitivity factor, the contribution of mass and structural changes can be obtained [39], [46]. Each point in the mass and anisotropy axis can be expressed by changes in the original coordinates (Δp and Δs) by the following equations:
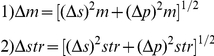



The sensitivity factor (S_f_), a measure of the sensitivity of the instrument for the *s*- pol relative to *p*-pol (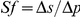), necessary to determine the mass and anisotropy axes has been determined, for the prism used in those experiments, to be 1.35 [46].

Affinities between the peptide and the lipids were obtained by plotting the PWR spectral changes that occur upon incremental additions of ligand to the cell. Since the PWR is only sensitive to the optical properties of material that is deposited on the resonator surface, there is no interference from the material that is in the bulk solution. Moreover, the amount of bound material is much smaller than the total amount of ligand present in the bulk solution, and it is assumed that the bulk material is able to freely diffuse and equilibrate with the membrane. Data fitting (GraphPad Prism) through a hyperbolic saturation curve provides the dissociation constants. It should be noted that since concomitantly with the binding process other processes, such as membrane reorganization and solvation, occur, the dissociation constants correspond to apparent dissociation constants. Kinetic experiments were performed, by following the PWR resonance minimum as a function of time. The data was fitted using exponential functions with GraphPad Prism.

### Isothermal titration calorimetry (ITC) measurements on whole cells

ITC experiments were performed on a TA Instrument Nano ITC calorimeter. The experiments were performed at 37°C. CHO cells (1.5×10^6^), resuspended in 1.5 mL 50 mM Tris-HCl, 100 mM NaCl and 6 mg/mL glucose, were filled into the microcalorimeter cell compartment and the peptide at the concentration of 1 mM or 155 µM into the syringe compartment. After reaching thermal equilibrium, 50 µL of 1 mM or 155 µM peptide solutions were added into the cell, leading to final extracellular concentrations of 32 µM or 5 µM respectively, and stirred constantly to avoid cell deposition in the bottom of the compartment. After the ITC measurements, a constant baseline was subtracted and the heat of interaction after injection plotted versus time. Two control experiments were performed: 50 µL of buffer was injected into the microcalorimetric compartment containing the cells; 50 µL of 1 mM or 155 µM peptide solution were injected into the microcalorimetric compartment that is containing buffer (no cells). The total heat signal was determined from the area underneath the curve (after subtracting the heats of dilution resulting from the control experiments) using the program NanoAnalyze provided by TA Instruments. The experiments were repeated 4 to 6 times for each cell line.
